# Effect of the ABCA1 agonist CS-6253 on amyloid-β and lipoprotein metabolism in cynomolgus monkeys

**DOI:** 10.1186/s13195-022-01028-1

**Published:** 2022-06-24

**Authors:** Sasan D. Noveir, Bilal E. Kerman, Haotian Xian, Cristiana Meuret, Sabrina Smadi, Ashley E. Martinez, Johannes Johansson, Henrik Zetterberg, Bryan A. Parks, Zsuzsanna Kuklenyik, Wendy J. Mack, Jan O. Johansson, Hussein N. Yassine

**Affiliations:** 1 Departments of Medicine and Neurology, University of Southern California, Los Angeles, CA 90033 USA; 2 Artery Therapeutics, Inc., San Ramon, CA 94583 USA; 3 Department of Psychiatry and Neurochemistry, Institute of Neuroscience and Physiology, the, Sahlgrenska Academy at the University of Gothenburg, Mölndal, Sweden; 4 Clinical Neurochemistry Laboratory, Sahlgrenska University Hospital, Mölndal, Sweden; 5 Department of Neurodegenerative Disease, UCL Institute of Neurology, Queen Square, London, UK; 6 UK Dementia Research Institute at UCL, London, UK; 7 Hong Kong Center for Neurodegenerative Diseases, Hong Kong, China; 8 Centers for Disease Control and Prevention, Atlanta, GA 30341 USA; 9 Department of Population and Public Health Sciences, University of Southern California, Los Angeles, CA 90033 USA

**Keywords:** ABCA1, Apolipoprotein E, Alzheimer’s disease, CS-6253

## Abstract

**Background:**

Inducing brain ATP-binding cassette 1 (ABCA1) activity in Alzheimer’s disease (AD) mouse models is associated with improvement in AD pathology. The purpose of this study was to investigate the effects of the ABCA1 agonist peptide CS-6253 on amyloid-β peptides (Aβ) and lipoproteins in plasma and cerebrospinal fluid (CSF) of cynomolgus monkeys, a species with amyloid and lipoprotein metabolism similar to humans.

**Methods:**

CS-6253 peptide was injected intravenously into cynomolgus monkeys at various doses in three different studies. Plasma and CSF samples were collected at several time points before and after treatment. Levels of cholesterol, triglyceride (TG), lipoprotein particles, apolipoproteins, and Aβ were measured using ELISA, ion-mobility analysis, and asymmetric-flow field-flow fractionation (AF4). The relationship between the change in levels of these biomarkers was analyzed using multiple linear regression models and linear mixed-effects models.

**Results:**

Following CS-6253 intravenous injection, within minutes, small plasma high-density lipoprotein (HDL) particles were increased**.** In two independent experiments, plasma TG, apolipoprotein E (apoE), and Aβ42/40 ratio were transiently increased following CS-6253 intravenous injection. This change was associated with a non-significant decrease in CSF Aβ42. Both plasma total cholesterol and HDL-cholesterol levels were reduced following treatment. AF4 fractionation revealed that CS-6253 treatment displaced apoE from HDL to intermediate-density- and low density-lipoprotein (IDL/LDL)-sized particles in plasma. In contrast to plasma, CS-6253 had no effect on the assessed CSF apolipoproteins or lipids.

**Conclusions:**

Treatment with the ABCA1 agonist CS-6253 appears to favor Aβ clearance from the brain.

**Supplementary Information:**

The online version contains supplementary material available at 10.1186/s13195-022-01028-1.

## Introduction

Deposition of extra-cellular amyloid-β peptide (Aβ) plaques in the brain is a feature of Alzheimer’s disease (AD) pathology as Aβ monomers can aggregate, which form fibrils and senile plaques [1–3]. The ratio of Aβ42 to Aβ40 in plasma is a promising biomarker for selecting patients with brain amyloid accumulation. Low plasma Aβ42/40 ratio has been associated with increased risk of dementia, more pronounced decline in cognitive function, and increased fibrillary Aβ deposition in the brain [4–6]. Aβ plaque brain deposition has been linked to cholesterol and lipid metabolism, both in the brain and in the periphery. In the brain, neuronal production of Aβ is controlled by membrane cholesterol content. Cholesterol content of neurons is kept low, inhibiting Aβ accumulation [7]. Aβ is cleared from the brain into the peripheral circulatory system. Sequestration of Aβ by sLRP [8] or by Aβ antibodies [9] in the periphery can promote Aβ efflux from the brain. In plasma, Aβ also interacts with apolipoprotein E (apoE), apolipoprotein A-I (apoA-I), or apolipoprotein C-III (apoC-III) [10]. Moreover, Aβ can interact with high-density lipoprotein (HDL) and very-low-density lipoprotein (VLDL) particles in the plasma and CSF [11].

ATP binding cassette 1 (ABCA1) participates in the formation of nascent HDL particles [12] and in the clearance of Aβ from the brain [13]. Recently, studies using CS-6253, an alpha-helical peptide designed from the C-terminus of apoE, to induce ABCA1 activity have shown promising results in reducing AD-related pathology in animal models [14]. With a greater binding affinity to ABCA1 than apoE, CS-6253 prevents ABCA1 degradation by stimulating ABCA1 recycling to the cell membrane which is associated with augmented cholesterol efflux to primarily apoE acceptor particles [15, 16]. Consistent with ABCA1 regulating lipidation of apoE, treatment of apoE4-targeted replacement (ApoE4-TR) mice with the ABCA1 agonist, CS-6253, increased apoE4 lipidation. This was accompanied by a reversal of apoE4-related cognitive and brain pathologies, including intraneuronal Aβ42 accumulation [14] and was associated with an increase in plasma apoE concentrations [17]. Furthermore, in a similar model, CS-6253 decreased apoE4 and ABCA1 aggregation in hippocampal homogenates of ApoE4-TR mice [16], supporting the importance of apoE lipidation in preventing its aggregation.

The effect of CS-6253 on plasma and CSF lipoproteins, together with measures of Aβ in primates, have not yet been studied. We hypothesized that treatment with CS-6253 by virtue of inducing ABCA1 activity would influence lipoprotein dynamics, including that of apoE particles, to promote Aβ clearance. We tested this hypothesis in monkeys, as part of the CS-6253 IND-enabling toxicology studies, in three cynomolgus monkey studies: the preliminary pharmacokinetics (PK) assessment study, the 10-day non-Good Laboratory Practice (GLP) dose-range finding (DRF) study, and the 30-day GLP study.

## Methods

### Study designs

The preliminary PK study included 2 male cynomolgus monkeys, each injected intravenously with a single dose of 25 mg/kg CS-6253. Blood samples were taken pre-injection, baseline, and at 5 min, 30 min, 1 h, 2 h, 4 h, 6 h, 12 h, 24 h, 48 h, and 72 h post-injection (p.i.). One aliquot of CSF was collected at baseline and 6 h after injection. The DRF study included three active CS-6253 groups: 75 mg/kg (low-dose), 150 mg/kg (mid-dose), and 225 mg/kg (high-dose) and a placebo group. Each of the 4 groups contained 2 male cynomolgus monkeys and 2 female cynomolgus monkeys. Monkeys were dosed every other day for a total of 5 times and blood samples were taken at baseline (2 weeks pre-injection), and at 10 min, 2 h, 4 h, 12 h, 24 h, and 48 h post-injection on days 1 and 9. Baseline measurements were used for normalization of the measurements. CSF was collected at 6 h after the last dose, at day 9. In the 30-day GLP dosing study CS-6253 10 mg/kg (low-dose), 25 mg/kg (mid-dose), and 75 mg/kg (high-dose) was injected every other day (QAD) for 28. The CS-6253 75 mg/kg high-dose and placebo groups consisted of 10 (5 male and 5 female) cynomolgus monkeys each. The CS-6253 10 mg/kg and 25 mg/kg groups consisted of 6 (3 male and 3 female) monkeys each. Animals were terminated 2 days after the last injection. Blood samples were collected at 5 min, 2 h, 4 h, 12 h, 24 h, and 48 h p.i. on days 1, 9, and 25. The measurements were normalized to the first reading that was taken at 5 min after the first injection. See also Table S1 for details of each study. All experimental procedures were conducted according to the approved protocols from the relevant institutions: PK study, BTS research, OWAL Assurance ID: D16-00,768 (A4519-01)—IACUC: 19–023; DRF study, BASI / Inotiv, OWAL Assurance ID: D16-00,571 (A4058-01)—IACUC: 03-MK-2019; GLP study, Altasciences, OWAL Assurance ID: D16-00,639 (A4261-01)—IACUC: 147,820–01. The experiments at USC were approved by IACUC, protocol #21,225.

### CSF collection

CSF samples were collected from the monkeys in the PK and DRF studies. In short, following standard procedures animals were anesthetized with intramuscular injection of ketamine and dexmedetomidine for the procedure. CSF was collected aseptically by cisterna magna puncture from all animals. Intramuscular atipamezole was administered as a reversal agent for dexmedetomidine after the procedure. CSF samples were divided into aliquots and frozen at 70 °C until shipped on dry ice by overnight delivery to the biomarker laboratory for analysis.

### CS-6253 concentration analysis

CSF and plasma CS-6253 levels were assayed using an ultra-high performance liquid chromatography (UHPLC) with tandem mass spectrometry (MS/MS) bioanalytical method at Climax (San Jose, CA, USA).

### Amyloid-related measurements in plasma and CSF

Plasma and CSF were collected and Aβ42 and Aβ40 concentrations were measured by sensitive Single molecule array (Simoa) technique (Quanterix Corp., Billerica, MA, USA). Concentrations of APP and AP2B1 were measured using a targeted mass spectrometry method, as previously described [18].

### ApoE measurements in plasma

DRF study plasma samples were diluted 1:5000 and GLP study plasma samples were diluted 1:15,000. ApoE levels were measured using Sandwich ELISA. The readings were analyzed using Myassays Four Parametric Logistic Curve. Note that plasma apoE levels for the 75 mg/kg dose in the GLP study were not measured.

### Plasma triglyceride, cholesterol, and pre-β-HDL measurements

Plasma triglyceride levels in the DRF study were measured using the L-Type Triglyceride M test (Fujifilm) according to the manufacturer’s instructions. Samples were diluted three times before the measurement. Total cholesterol levels in plasma were measured using Cholesterol E kit (Fujifilm). HDL cholesterol levels were measured using the HDL-Cholesterol E kit (Fujifilm). Plasma triglyceride, HDL, LDL, and total cholesterol levels in the GLP study samples were measured by IDEXX Laboratories. Data was analyzed using linear quantification. The plasma from the monkeys in the PK study were diluted 1:50 and pre-Beta HDL levels were measured using pre-β1 HDL ELISA kits (Daiichi Pure Chemicals, Inc.) according to the manufacturer’s instructions. The data was analyzed using Myassays Four Parametric Logistic Curve.

### Plasma dextran sulfate lipoprotein preparation

In a 96-well round-bottom plate compatible with an accompanying magnetic separator (EpiGentek, Cat. # Q10002-1), an aliquot (30 μL) of plasma from each timepoint was mixed with 70 μL of a primary precipitating solution, then incubated on ice for 15 min. The samples were then centrifuged (2000 RCF, 10 min, 4 °C). The resultant supernatants were mixed in equal proportion with a secondary precipitating solution containing dextran sulfate and incubated at room temperature for 3 min. A volume of 20 μL of magnetic beads (Sigma, Cat. # GE24152105050250**)** solution $\left[3.5\frac{\mathrm{mg}}{\mathrm{ml}}\right]$ was added to each suspension, then incubated at room temperature for 3 min. The beads were washed with MQ water (60 μL × 2) following a magnetic pulled down. The beads were then washed with 30 μL of releasing buffer (× 2), subjected to a magnetic pulled down, and the supernatants were pooled for analysis.

### Ion-mobility analysis

Monkey plasma samples treated with dextran sulfate were introduced into a charge-reducing electrospray (TSI Inc., model 3482) every 13 min by automated loop injections via an integrated autosampler (Teledyne CETAC Technologies, model MVX-7100). Electrospray settings were as follows: voltage 2.0 kV, CO_2_ flow 0.15 slmp, and airflow 1.5 slmp. The differential mobility analyzer (TSI Inc., model 3085), coupled to a condensation particle counter (TSI Inc, model 3788), scanned particles 4.45 to 63.8 nm for 180 s. The generated data of interest was analyzed on Fityk (version 1.3.1), as previously described, and graphed using OriginPro software (version 2021). Voigt probability distribution curves were generated from particle count (#/mL) vs diameter range for lipoprotein subclasses and normalized by dividing sub-classes with the sum of peak areas from all lipoproteins present within the spectrum.

### Isolation and examination of CSF and plasma lipoprotein fractions using AF4, MRM, and DLS

CSF and plasma samples from the DRF study were sent to the CDC Division of Laboratory Sciences. 50 μL of each plasma sample, at three time points (5 min, 4 h, and 12 h) from two monkeys each, was injected into the asymmetric-flow field-flow fractionation (AF4) system, collecting a set of 40 fractions from each sample. The fractions and all unfractionated CSF and plasma were analyzed by three LC–MS/MS methods using multiple reaction monitoring (MRM) as described elsewhere [19–21], quantifying proteins typically detected in HDL subclasses, main non-polar lipids (free cholesterol and cholesteryl ester), and phospholipid classes (PC, SM, LPC, PE, and PI). Particle sizes in the fractions were determined using dynamic light scattering (DLS) as previously described [22]. The moles of analytes in the sized fractions were divided by the volume of plasma injected into the AF4 channel, giving equivalent analyte concentrations in plasma.

#### Statistical analysis

We used multiple linear regression models and linear mixed-effects models to analyze the effects of CS-6253 over time on levels of various biomarkers (baseline-normalized on a percentage scale with baseline values of 100%). Multiple linear regression models were used on those datasets from the DRF study with only two CSF measurement time points per monkey subject. These models were fitted using ordinary least squares estimation. In each model, the main CSF biomarker outcome of interest was modeled as a function of dose (with placebo dose indicated as 0) and timepoint (endpoint compared with baseline measurement), with an interaction term of dose and timepoint; a significant interaction term indicates a mean difference in the biomarker change compared with placebo. Linear mixed-effects models were used on datasets from both the DRF and GLP studies, where each monkey subject had repeated plasma measurements throughout the study. These mixed-effects models were fitted using restricted maximum likelihood estimation. In some of the mixed-effects models, the main plasma biomarker outcome of interest (cholesterol, triglycerides, apoE) was modeled as a function of fixed effects including treatment (active compared with placebo), and indicator variables for hours since injection (i.e., time of injection) and injection number, and total time under study; a random intercept of the subject was specified to model correlated outcomes arising from repeated measurements. Since Aβ40 and Aβ42 measurements were not obtained in placebo-treated monkeys at all time points at which treated monkeys were assessed, the mixed-effects models for these measurements (and the Aβ42/40 ratio) used only actively treated animals; fixed effects included indicator variables for treatment dose, injection number, and time of assessment (4 h and 48 h, each compared with the 5-min baseline timepoint). All data were standardized as needed to obtain standardized parameters. Wald approximation was used to obtain p-values and confidence intervals. A 2-sided *p* value of less than 0.05 was considered statistically significant. All models were evaluated for assumptions of normality and homoscedasticity using residual plots. Statistical analyses were conducted using the lme4 package in R version 4.0.5.

For some of the variables, which showed a treatment-related trend without statistical significance, we performed a sample size calculation analysis using the pwr package in R to determine the sample sizes needed to detect a certain effect size in each of those variables (Table S2). All calculations were done with a two-sample independent *t*-test, with power set at 0.8 and significance level alpha set at 0.05 and using a one-sided form of the alternative hypothesis. Cohen’s effect size, d was calculated as

$$d=\frac{|{X}_{1}-{X}_{2}|}{SD}$$

where *X* is the mean value of each sample groups and SD is the pooled standard deviation of the two groups.

## Results

### Increase in plasma amyloid-β 42/40 ratio following treatment with CS-6253

**Fig. 1:**
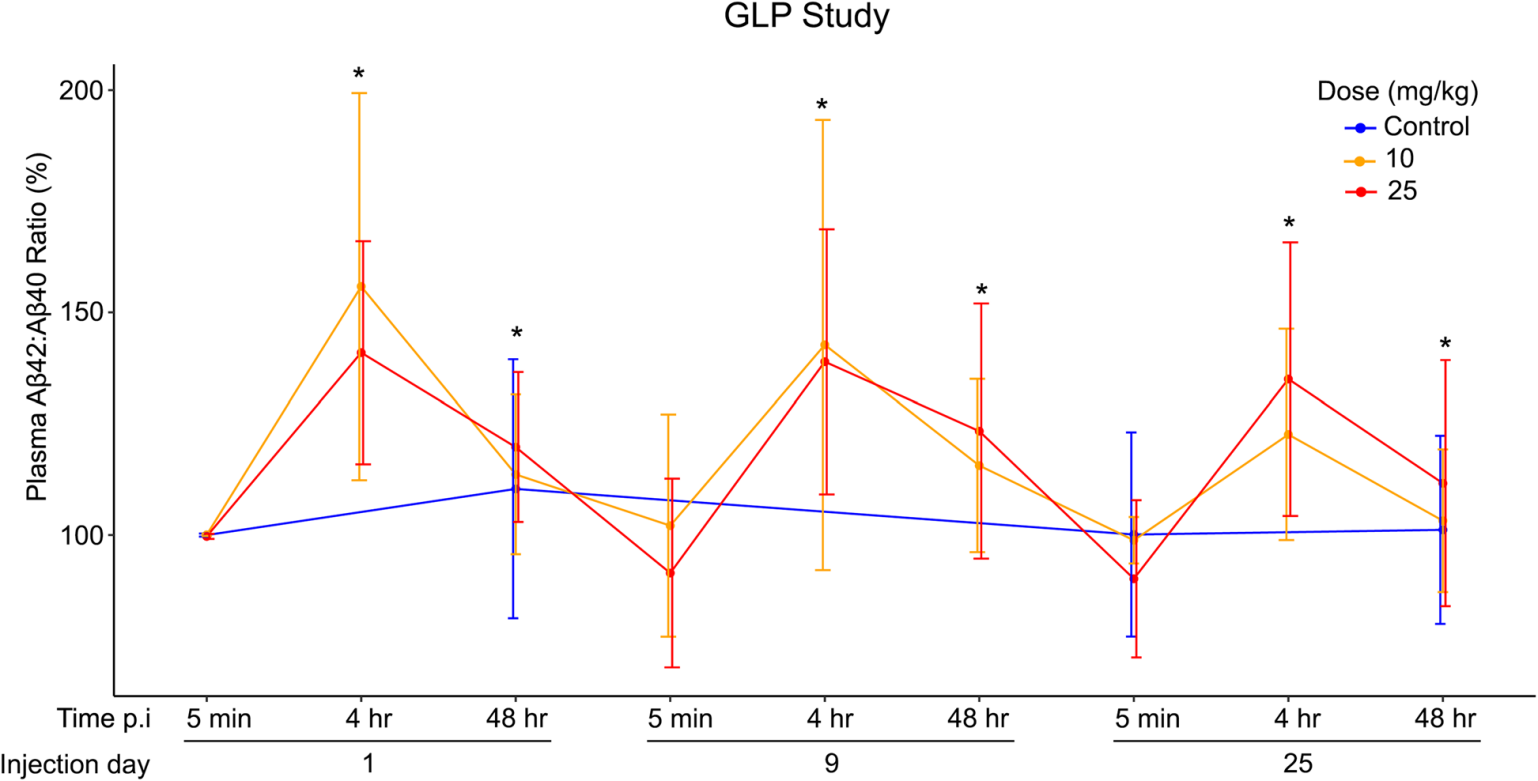
Plasma Aβ42/40 levels were increased following CS-6253 injection in the GLP study. The increase at 4 h and 48 h p.i. compared with 5 min p.i. on the day of injection were statistically significant (*p* < 0.001 for both). The values were shown as percent change from the first measurement point at 5 min p.i. on day 1. The measurements in the placebo arm were only done at 5 min and 48 h on days 1 and 25. The mixed-effects models for this measurement used only actively treated animals; fixed effects included indicator variables for treatment dose, injection number, and time of assessment (4 h and 48 h, each compared with the 5-min baseline timepoint). A random intercept of subject was specified to model correlated outcomes arising from repeated measurements

In the case of the 30-day GLP study, an increase in the plasma Aβ42/40 ratio following injection of CS-6253 was observed (Fig. 1). The change was statistically significant at 4 h and 48 h post-injection (p.i.) compared with the Aβ42/40 ratios 5 min p.i. to account for the effect of CS-6253 injection over time (Fig. 1). Both Aβ42 and Aβ40 levels in plasma were lower in the treatment groups compared with controls with a more pronounced decrease for Aβ40, resulting in the observed increase in the plasma Aβ42/40 ratio (Fig. S1). In the case of the DRF study, Aβ42 and Aβ40 levels increased in plasma 6 h p.i. compared with pre-treatment (Fig. S2A-B). The Aβ42/40 ratio in the treatment groups also increased compared with the controls after 6 h p.i. on day 9 (Fig. S2C). Due to methodological differences, the measurements in the two studies could not be compared directly. In the DRF study, changes after treatment were normalized to the baseline, whereas in the GLP studies, the changes were normalized to 5 min p.i. Additionally, the time intervals between measurements and the dosing vary between the two studies. Yet, consistent trends were observed in both studies. Taken together, these findings suggested that CS-6253 transiently increased the plasma Aβ42/40 ratio within 4 to 6 h p.i.

### CS-6253 brain penetrance

CS-6253 concentrations in CSF were assessed by LC–MS/MS 6 h after dosing in the preliminary PK study (CS-6253 25 mg/kg single dose IV) and 6 h after the 5th/last dose in the DRF study (Placebo vs CS-6253 75, 150 and 225 mg/kg). In both studies, the CSF/plasma-ratio at the 6-h time point was < 1%.

### Changes in CSF Aβ and lipoprotein levels following treatment with CS-6253

Interestingly, the Aβ42/40 ratio in the CSF of monkeys in the DRF study did not change significantly (Fig. S2F), despite accompanied by a dose–response but a non-significant decrease in both Aβ42 and Aβ40 levels (Fig. S2D-E). Moreover, Amyloid-β precursor protein (APP) levels in the CSF of monkeys in the DRF study had a noticeable but statistically non-significant decrease after CS-6253 treatment that is likely resulting from the small sample size (Fig. S2G; See Table S2 for sample size calculations). The change in APP levels, however, correlated with CSF Aβ42 level changes (Fig. S2H). Previously, endo-lysosomal protein AP2B1 was found to increase in AD patients’ CSF [18]. In our study, similarly to APP, there was a lowering dose–response trend for CSF AP2B1 levels after CS-6253 treatment, but the results were not statistically significant (Fig. S2I), also likely due to the small sample size. This change in CSF AP2B1 levels correlated directly with changes in Aβ42 levels (Fig. S2J). CSF apolipoprotein and lipid levels did not change after treatment (Fig. S3).

### Increase in plasma apoE following treatment with CS-6253

**Fig. 2:**
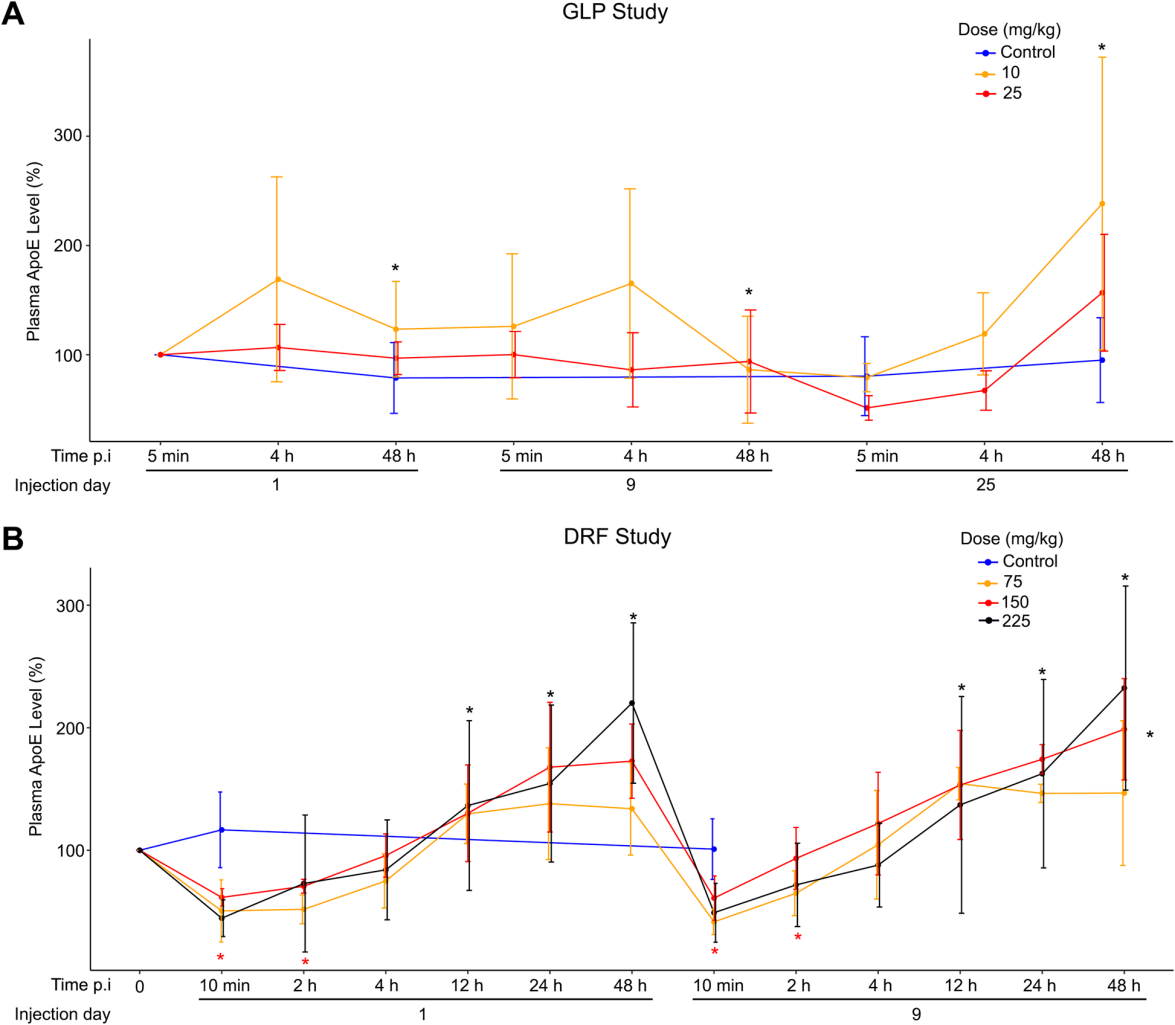
Plasma apoE levels increased overtime in both studies. **A** In the GLP study, CS-6253 treatment significantly increased plasma apoE levels 48 h p.i. (*p* = 0.008). **B** In the DRF study, at 10 min and 2 h p.i. apoE decreased significantly (*p* < 0.001 and *p* = 0.020, respectively). Then, apoE significantly increased at 12 h, 24 h, 48 h p.i. (*p* < 0.001 for all 3) and accumulated treatment over time (*p* = 0.040). The values for the GLP study were shown as percent change from the first measurement point at 5 min p.i. on day 1 while the values for the DRF study were shown as percent change from the baseline measurement. The analysis was done using a mixed-effects model, with apoE modeled as a function of fixed effects including treatment (active compared with placebo), indicator variables for hours since injection (i.e., time of injection), and a linear variable for total time under study; a random intercept of subject was specified to model correlated outcomes arising from repeated measurements

CS-6253 treatment increased apoE levels in the plasma of monkeys in the GLP study. This effect was statistically significant at 48 h p.i. compared to 5 min p.i. (Fig. 2A). In the DRF study with the higher CS-6253 doses, the results were more complex. Initially, apoE levels decreased some in the plasma after CS-6253 treatment compared to the baseline but starting at 12 h p.i., apoE increased significantly (Fig. 2B). Overall, CS-6253 treatment increased apoE levels in the plasma. In the PDAPP transgenic mouse model of AD, ApoE4-TR mice had lower CSF and plasma levels of apoE compared with ApoE2-TR and ApoE3-TR mice accompanied with increased amyloid deposition in the brain [23]. Similarly, plasma, but not CSF, apoE levels were lower in apoE4 carriers compared with non-carriers [24], and lower apoE in plasma is associated with increased AD risk [25–27]. Thus, increasing plasma apoE levels via CS-6253 may have therapeutic benefits in AD.

### Changes in plasma lipid levels following treatment with CS-6253

**Fig. 3:**
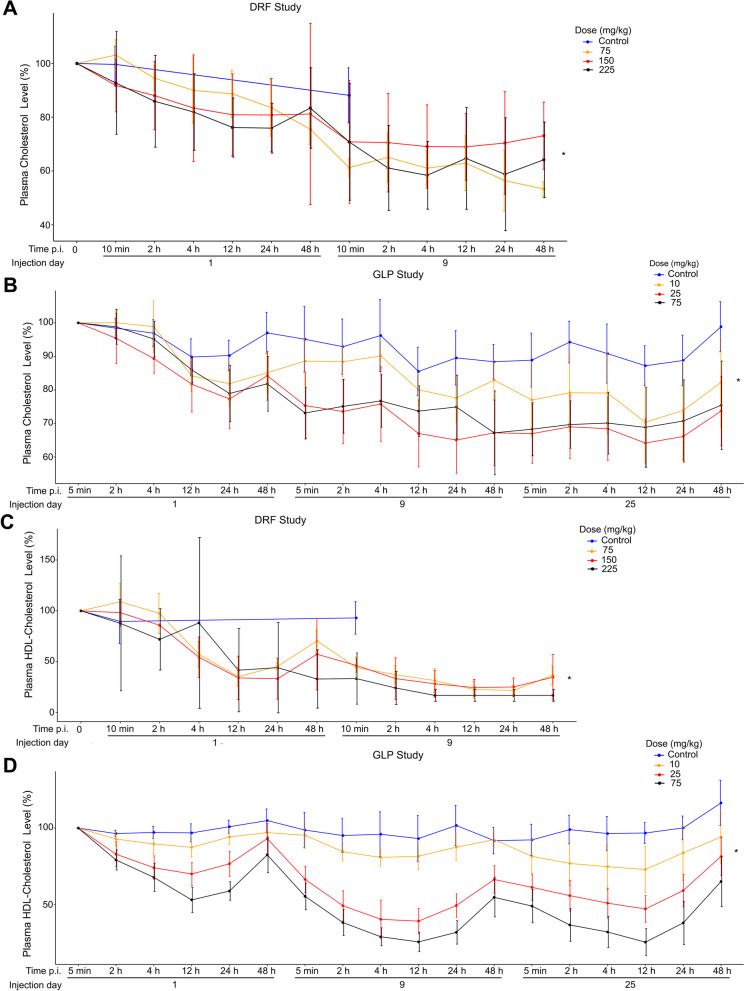
Plasma total cholesterol and HDL-cholesterol levels decreased after CS6253 treatment. **A** In the DRF study, decrease in plasma total cholesterol levels was significant at 2 h, 4 h, 12 h, 24 h, and 48 h p.i. (*p* = 0.002, *p* < 0.001, *p* < 0.001, *p* < 0.001, *p* = 0.001, respectively) and for accumulated treatment over time (*p* < 0.001). **B** In the GLP study, decrease in plasma total cholesterol levels was significant for treatment and for accumulated treatment over time (*p* < 0.001 for both), as well as at 12 h, 24 h, and 48 h p.i. (*p* < 0.001, *p* < 0.001, and *p* = 0.025, respectively). **C** In the DRF study, decrease in plasma HDL cholesterol levels was significant for 2 h, 4 h, 12 h, 24 h, and 48 h p.i. (*p* = 0.008, *p* < 0.001, *p* < 0.001, p < 0.001, *p* < 0.001, respectively) and for accumulated treatment over time (*p* < 0.001). **D** In the GLP study, decrease in plasma HDL cholesterol levels was significant for treatment and for accumulated treatment over time (*p* < 0.001 for both). Additionally, the time after injection was significantly correlated with HDL cholesterol levels in a quadratic model (*p* < 0.001). The values for the DRF study were shown as percent change from the baseline measurement while the values for the GLP study were shown as percent change from the first measurement point at 5 min p.i. on day 1. The analysis was done using a mixed-effects model, with cholesterol or HDL cholesterol levels modeled as a function of fixed effects including treatment (active compared with placebo), indicator variables for hours since injection (i.e., time of injection), and a linear variable for total time under study; a random intercept of subject was specified to model correlated outcomes arising from repeated measurements

Induction of cholesterol efflux from macrophages by activation of ABCA1 is rate-limiting for reverse cholesterol transport, i.e. the efflux of excess cholesterol from peripheral tissues to be transported via plasma to the liver for biliary excretion [28]. Excess cholesterol synthesized in the brain is also removed into the periphery through plasma [29]. Thus, we analyzed if CS-6253 treatment affected cholesterol levels in the plasma. CS-6253 significantly decreased plasma cholesterol levels in both DRF and GLP Studies (Fig. 3A and B). This decrease in total cholesterol (total-C) levels was also reflected by a decrease in HDL-cholesterol (HDL-C) levels (Fig. 3C and D). Interestingly, the HDL-C levels followed a periodic pattern decreasing after injection and increasing back at 48 h p.i. (Fig. 3D). Taken together, these results suggested that CS-6253 treatment lowered both total-C and HDL-C in plasma. The relationship between cholesterol-lowering drugs and AD is complicated. While in some studies lowering plasma cholesterol levels via statins or other interventions reduced AD risk, in others it had no effect [30, 31]. Regardless, in line with our findings, many in vitro and in vivo studies showed that lowering cholesterol is associated with increased apoE and decreased Aβ deposition [30, 31].

Additionally, we analyzed the plasma HDL-C/apoA-I ratio, an indicator of HDL particle size, after CS-6253 treatment in the GLP study. It has been shown that small particles distribute more in the extra-vascular space than larger particles [32], which may be of interest for tissue penetration of apoA-I, a natural ABCA1 agonist. A significant decrease in the HDL-C/apoA-I ratio was observed at 4 h p.i. compared with 5 min p.i. which increased later (Fig. S4). Thus, CS-6253 transiently decreased plasma HDL-C/apoA-I ratio for a short period. Interestingly, treatment groups in the GLP study did not appear to have significant differences in plasma LDL cholesterol levels compared with the control group (Fig. S5). We did not observe significant changes in other lipids or apolipoproteins in the plasma (Fig. S6).

**Fig. 4:**
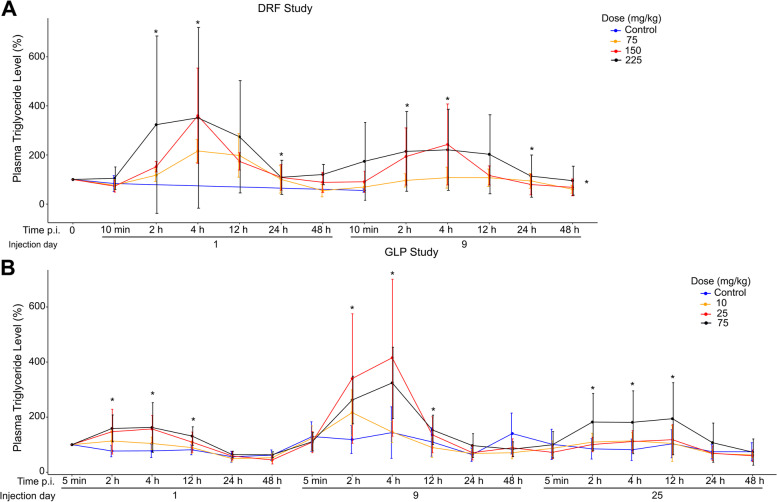
Plasma triglyceride levels increased after CS-6253 treatment. **A** In the DRF study, increase in plasma triglyceride levels was significant at 2 h, 4 h, and 12 h p.i. (*p* = 0.002, *p* < 0.001, and *p* = 0.003, respectively) and for accumulated treatment over time (*p* = 0.016). **B** Increase in plasma triglyceride levels in treated monkeys was significant at 2 h, 4 h, and 12 h p.i. in the GLP study (*p* < 0.001, *p* < 0.001, and *p* = 0.050, respectively). The values for the DRF study were shown as percent change from the baseline measurement while the values for the GLP study were shown as percent change from the first measurement point at 5 min p.i. on day 1. The analysis was done using a mixed-effects model, with triglyceride levels modeled as a function of fixed effects including treatment (active compared with placebo), indicator variables for hours since injection (i.e., time of injection) and injection number, and total time under study; a random intercept of subject was specified to model correlated outcomes arising from repeated measurements

Besides cholesterol, apolipoprotein particles carry triglycerides as a part of the tissue lipid homeostasis [33]. Therefore, we analyzed CS-6253’s effect on plasma triglyceride levels. The triglycerides in plasma increased significantly following CS-6253 injection and waned at about 24 h p.i. in the DRF study (Fig. 4A). This peak in triglycerides was reproduced in the GLP study, although it was less pronounced (Fig. 4B). Thus, continued CS-6253 treatment can maintain increased plasma triglyceride levels. Increase in triglycerides may reflect increased plasma apoE levels (Fig. 2). This effect might have been especially more pronounced in cynomolgus monkeys, whose apoE is more similar to human apoE4 than the other alleles [34], because apoE4 has higher affinity for VLDL [33].

### Analysis of pre-β HDL and s-, m-, and l-HDL particles

**Fig. 5:**
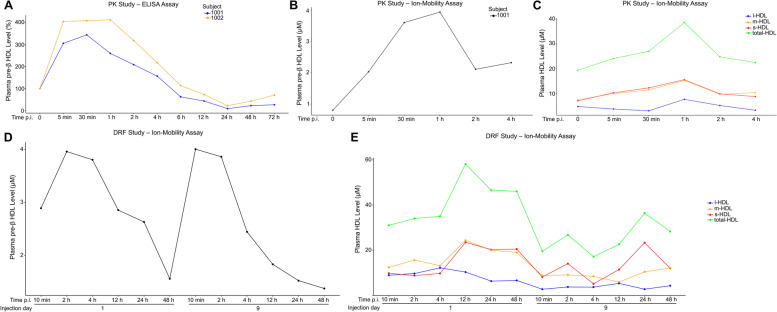
Analysis of pre-β HDL and s-, m-, and l-HDL particles in plasma. **A** Plasma pre-β HDL levels in the PK study were measured by ELISA assay (*n* = 2). **B** Plasma pre-β1 HDL levels were determined using ion-mobility analysis and Voigt probability distribution (see also Fig S7) for one monkey in the PK study. **C** For the same monkey, plasma s-, m-, and l-HDL particle concentrations were calculated using ion-mobility analysis and Voigt probability distribution. **D** For one monkey in the DRF study, plasma pre-β HDL levels were determined using ion-mobility analysis and Voigt probability distribution. **E** For the same monkey, plasma s-, m-, and l-HDL particle concentrations were calculated using ion-mobility analysis and Voigt probability distribution

Furthermore, we analyzed pre-β HDL and small- (s-), medium- (m-), and large- (l-) HDL particle numbers in plasma in the PK and DRF studies. To extract pre-β HDL and s-, m-, and l-HDL particle concentrations from the calibrated ion-mobility analysis, the concentration vs. size profiles were deconvoluted into Voigt probability distribution peaks. In addition, the baseline data was compared with published concentrations (Fig. S7, Table S3) [35]. The calculated size distribution and range for each particle are given in Fig. S7B. Pre-β HDL is a natural ABCA1 agonist and lipidation of pre-β HDL particles is one of the first steps in reverse cholesterol transport [36]. Pre-β HDL levels in plasma increased following CS-6253 injection but waned down as shown by an ELISA assay (Fig. 5A) and by ion-mobility analysis (Fig. 5B and D). The baseline s-, m-, and l-HDL particle concentrations in monkey plasma were similar to human plasma levels [35]. Total HDL particle concentrations for monkeys both in the DRF and the PK studies increased soon after injection and started going down (Fig. 5C and E). There was a general trend for decreasing l-HDL particle levels especially in the DRF study monkey plasma; thus, the change in total HDL was largely driven by changes in s- and m-HDL levels (Fig. 5C and E and Table S3). Moreover, the trend in decreasing l-HDL in the DRF study is in parallel to the decreasing HDL-C levels given that l-HDL particles are the major carriers of cholesterol [35, 37]. We also, analyzed IDL, LDL, Midzone, and VLDL particles for one sample in both studies (Fig. S8). The VLDL levels in the DRF study followed a similar trend to the plasma triglyceride levels in the same study (compare Fig. 4A and Fig. S8B). This trend is in line with previous findings [38].

### Shift of apoE from HDL to IDL/LDL particles

**Fig. 6:**
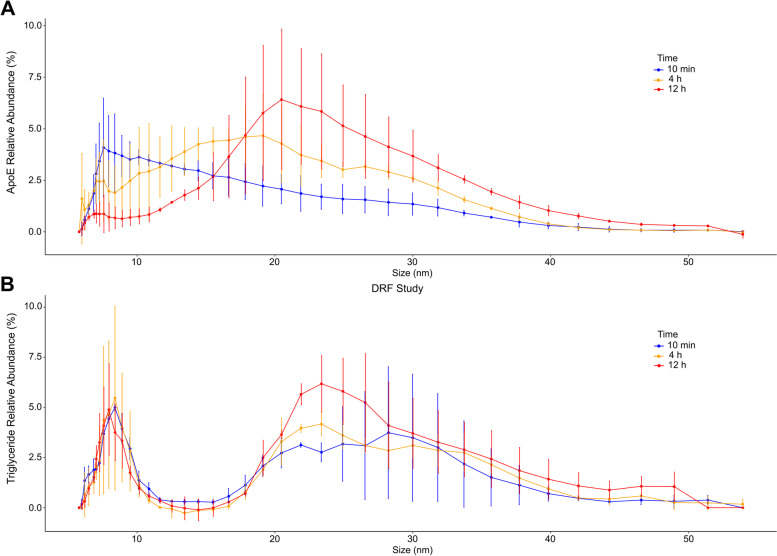
Analysis of abundance of apoE and triglycerides in HDL, IDL, and LDL particles using AF4 for two monkeys in the DRF study. **A** Following CS-6253 injection, apoE shifted to larger lipoprotein particles. **B** The triglycerides distribution among lipoproteins shifted towards IDL and LDL size

Finally, we analyzed how the relative distribution of apolipoproteins and lipids across particle sizes after CS-6253 injection using AF4. Total apoE and triglyceride levels, measured by mass spectrometry, followed a similar pattern to our earlier measurements with ELISA (compare Fig. S9A to Fig. 2B and Fig S9B to Fig. 4A). Interestingly, analysis of sized fractions revealed that at 10 min after CS-6253 injection, apoE was mainly on the s- and m-HDL particles (Fig. 6A) but apoE shifted to the LDL and IDL particles at 4 h p.i. and was mostly present in these larger particles at 12 h p.i. (Fig. 6A). In contrast, apoA-I’s abundance shifted to smaller-sized HDL particles (Fig. S10A). The relative distribution of other apolipoproteins analyzed including apoC-III was not changed among the fractions (Fig. S10B). Relative abundance of triglyceride in the LDL and IDL particles increased at 12 h p.i. (Fig. 6B) reflecting shift in apoE distribution, in line with increased triglyceride levels following increased apoE levels (Figs. 2B, 4A, and S9).

## Discussion

In this study, cynomolgus monkeys were treated with CS-6253 as part of IND-enabling studies and its effects on lipid metabolism and AD biomarkers were assessed in plasma and CSF.

Since aggregation of Aβ in the brain contributes to the pathogenesis of AD, Aβ-related biomarkers are used for selecting the prodromal stages of this neurodegenerative disease [39, 40]. Particularly, recent studies have identified lower plasma Aβ42/40 ratio as a predictor of brain amyloidosis [4–6]. Accordingly, plasma Aβ42/40 ratio were used to test the effectiveness of CS-6253. Indeed, treatment with CS-6253 increased the plasma Aβ42/40 ratio, suggesting CS-6253 was able to facilitate Aβ brain to plasma flux in cynomolgus monkeys. One mechanism for this observation is the shift of apoE to larger triglyceride-containing particles, absorbing Aβ from the CSF and transporting it to the liver for clearance [11]. These results are consistent with previous studies in apoE4-targeted replacement mice, which showed that CS-6253 can counteract Aβ42 accumulation in hippocampal neurons and improve behavioral deficits [14].

CS-6253 treatment did not have a significant effect on CSF lipoproteins or lipids, possibly due to its poor transport into the brain. CSF to plasma concentrations at 6 h post-dosing was < 1%. Even though these low CS-6253 levels may have direct ABCA1 effects in the CNS, the pharmacological effects of CS-6253 are interpreted to be indirect in nature. A possible explanation for these findings follows the Peripheral-Sink Hypothesis [41], which postulates that Aβ-binding ligands in the periphery sequester Aβ, promoting efflux of Aβ from the CSF to the periphery. This aligns with the present study’s finding that CS-6253 was able to simultaneously increase Aβ42 concentrations in plasma and decrease them in CSF. Studies have shown support for this hypothesis, showing that increasing peripheral Aβ antibodies and Aβ-binding lipoproteins increase Aβ efflux [8, 42] through LRP1 [43, 44]. As Aβ is highly lipophilic, the majority of Aβ40 and Aβ42 in the circulation are bound to lipoproteins, particularly triglyceride-rich lipoproteins (TRLs) [45, 46]. Since apoE plays an important role in lipoprotein association with Aβ, with apoE-containing human plasma lipoproteins able to absorb excess Aβ [11]. It is likely then that Aβ may cross into the periphery with an increase in plasma apoE in TRLs. Indeed, the present report found that CS-6253 consistently caused a transient increase in plasma apoE concentrations in TRL particles. The transient nature of the plasma apoE and Aβ42/40 ratio increase may be explained by liver uptake of Aβ42 containing apoE particles by apoE receptors such as LRP1 [47], thus forming a vector from the brain, then to plasma, and finally to the liver for degradation or excretion. Low plasma apoE levels are associated with increased risk of AD [25–27]. However, the association between apoE levels and dementia risk does not appear to be linear. Examination of a prospective cohort with 105,949 white individuals revealed that an increase in plasma apoE level from 2.5 to 5 mg/dL was associated with lower dementia risk but when apoE level exceeded 5 mg/dL, the association with dementia risk was inversed [48]. Very high levels of plasma apoE are associated with an increased vascular risk [49].

In the periphery, apoE plays an important role in reverse cholesterol transport [50, 51]. In plasma, both apoE and apoA-I receive cholesterol and phospholipids from the plasma membrane of peripheral cells, via ABCA1, a process most pronounced in monocyte-macrophage cells. This reverse cholesterol transport results in the formation of HDL particles, which transport excess cholesterol to the liver for secretion [52]. In addition to demonstrating vasoprotective functions, plasma HDL particles have been implicated in protection from AD [53, 54]. Higher levels of apoE-HDL have been shown to increase triglyceride levels by inhibiting displacement of hepatic lipase, an enzyme, which must be liberated to hydrolyze triglycerides [55, 56]. The increase of apoE and triglycerides found in the present study suggests there may have been a substantial rise in apoE-HDL levels, but further investigation is necessary. The cooperation between apoE and HDL also has an important role in Aβ clearance. It has been shown that injecting apoE into the periphery in the presence of reconstituted HDL promoted the transport of Aβ across bioengineered cerebral blood vessels [57]. This suggests that interactions between apoE and HDL have synergistic effects on the clearance of Aβ across vasculature.

Low plasma HDL cholesterol levels have been linked to greater cerebral Aβ deposition [58]. Intravenous administration of HDL has been shown to reduce soluble levels of Aβ in the brain [59]. Levels of plasma apoA-I and apoE, which are components of plasma HDL, are lower in AD patients [60–63]. Isolated apoA-I binds to Aβ peptide and can prevent Aβ-induced toxicity and Aβ aggregation [64, 65]. Furthermore, plasma lipoproteins have been linked to the transport and clearance of Aβ from the brain [66]. Interestingly, adding CS-6253 to plasma has been shown to displace apoA-I from alpha-HDL particles, and stimulate the formation of pre-β HDL [15]. CS-6253 mimics apoA-I’s ability to interact with ABCA1 to form functional, so-called nascent HDL particles that are actively remodeled in plasma [15]. The capacity of CS-6253 to compete with other apolipoproteins such as apoE remains to be delineated.

The results of the present study validate previous findings in vitro which demonstrate the ability of CS-6253 to induce formation of pre-β HDL in plasma [15]. Treatment with CS-6253 increased plasma pre-β levels as soon as 5 min following injection. Plasma pre-β plays an important role in reverse cholesterol transport, as it efficiently stimulates ABCA1-dependent cholesterol efflux. This study also found that CS-6253 decreased HDL-C levels, which may account for the decrease in total-C levels. While low levels of HDL-C have been associated with negative AD outcomes, recent studies suggest that HDL particle functionality is important, for example, through cholesterol efflux from macrophages by ABCA1 [58, 67]. It has been shown that plasma HDL-C concentrations divided by apoA-I concentrations may be a better alternative to HDL-C levels alone in predicting mortality outcomes [68]. This may provide more information about the quality of HDL, as HDL is a dynamic and heterogeneous particle. Particularly, this ratio is thought to represent the amount of cholesterol per HDL particle. Accordingly, lower HDL-C/apoA-I ratios would represent a higher number of lipid-poor HDL particles, which are better able to pick up cholesterol from peripheral tissues than cholesterol-rich HDL particles. Indeed, it has been shown that individuals with lower HDL-C/apoA-I ratios had a decreased likelihood of subclinical atherosclerosis and mortality [68]. The present study found that when accounting for apoA-I, CS-6253 was able to decrease the HDL-C/apoA-I ratio, suggesting CS-6253 increases lipid-poor HDL particles. Interestingly, the time of the HDL-C/apoA-I decrease (4 h after treatment) correlates with the time of the plasma Aβ42/40 ratio increase. The significance of this is unclear and more work needs to be done to understand the plasma HDL-C/apoA-I in relation to AD. It is possible that exchangeable apolipoproteins such as apoE and apoA-I which are present on lipid-poor s-HDL may enter the brain and become lipidated via ABCA1 [8, 69, 70]. This may allow for the transport of brain lipids and peripheral lipoproteins, which are important for Aβ clearance from the brain. However, we did not detect any changes in CSF lipids or apolipoproteins in this study.

### Limitations

This study has some limitations. The findings presented do not show direct activation of ABCA1 in cynomolgus monkeys and therefore might not be directly related to reverse cholesterol transport. For example, CS-6253 treatment effects may not be on macrophages, but on liver or other cells. The study design precludes such an examination. Second, Aβ peptides were not measured on any lipoprotein fractions given the low abundance of Aβ peptides and a limited amount of samples available in this study. In addition, it remains to be demonstrated whether pre-beta HDL formed from CS-6253 or apoE containing TG-rich particles, or both are driving the clearance Aβ peptides with vasoprotective or AD protective effects. Finally, the small sample sizes, particularly for CSF studies, explain why the changes in CSF Aβ levels after treatment did not reach statistical significance.

## Conclusions

Despite these limitations, the findings reported here support that treatment with the ABCA1 agonist CS-6253 in cynomolgus monkeys can lead to favorable AD biomarkers changes that, if confirmed in ensuing human studies, will allow the generation of useful biomarkers to guide CS-6253 drug development into the AD space.

## Supplementary Information

**Additional file 1**. 

## Data Availability

Data sharing is not applicable to this article as no datasets were generated or analyzed during the current study. All other data are available from the corresponding author on reasonable request.

## Abbreviations

ABCA1: ATP binding cassette 1
AD: Alzheimer’s disease
Aβ: Amyloid-β peptides
APP: Amyloid-β precursor protein
AF4: Asymmetric-flow field-flow fractionation
apoA-I: Apolipoprotein A-I
apoC-III: Apolipoprotein C-III
apoE: Apolipoprotein E
ApoE4-TR: ApoE4-targeted replacement mice
CSF: Cerebrospinal fluid
DLS: Dynamic light scattering
DRF: Dose-range finding
GLP: Good Laboratory Practice
HDL: High-density lipoprotein
s-HDL: Small-HDL
m-HDL: Medium-HDL
l-HDL: Large-HDL
HDL-C: HDL-cholesterol
IACUC: Institutional Animal Care and Use Committee
IDL: Intermediate-density lipoprotein
MRM: Multiple reaction monitoring
LDL: Low-density lipoprotein
p.i.: Post-injection
PK: Pharmacokinetics
TG: Triglyceride
total-C: Total-cholesterol
TRLs: Triglyceride-rich lipoproteins
VLDL: Very-low-density lipoprotein
