# Supplementary material for: Effect of the ABCA1 agonist CS-6253 on amyloid-β and lipoprotein metabolism in cynomolgus monkeys
Source: Alzheimers Res Ther. 2022 Jun 24;14:87. doi: 10.1186/s13195-022-01028-1 (PMC9229758; doi:10.1186/s13195-022-01028-1)
Supplement: Supplementary file 1 — Additional file 1. [file 13195_2022_1028_MOESM1_ESM.docx]

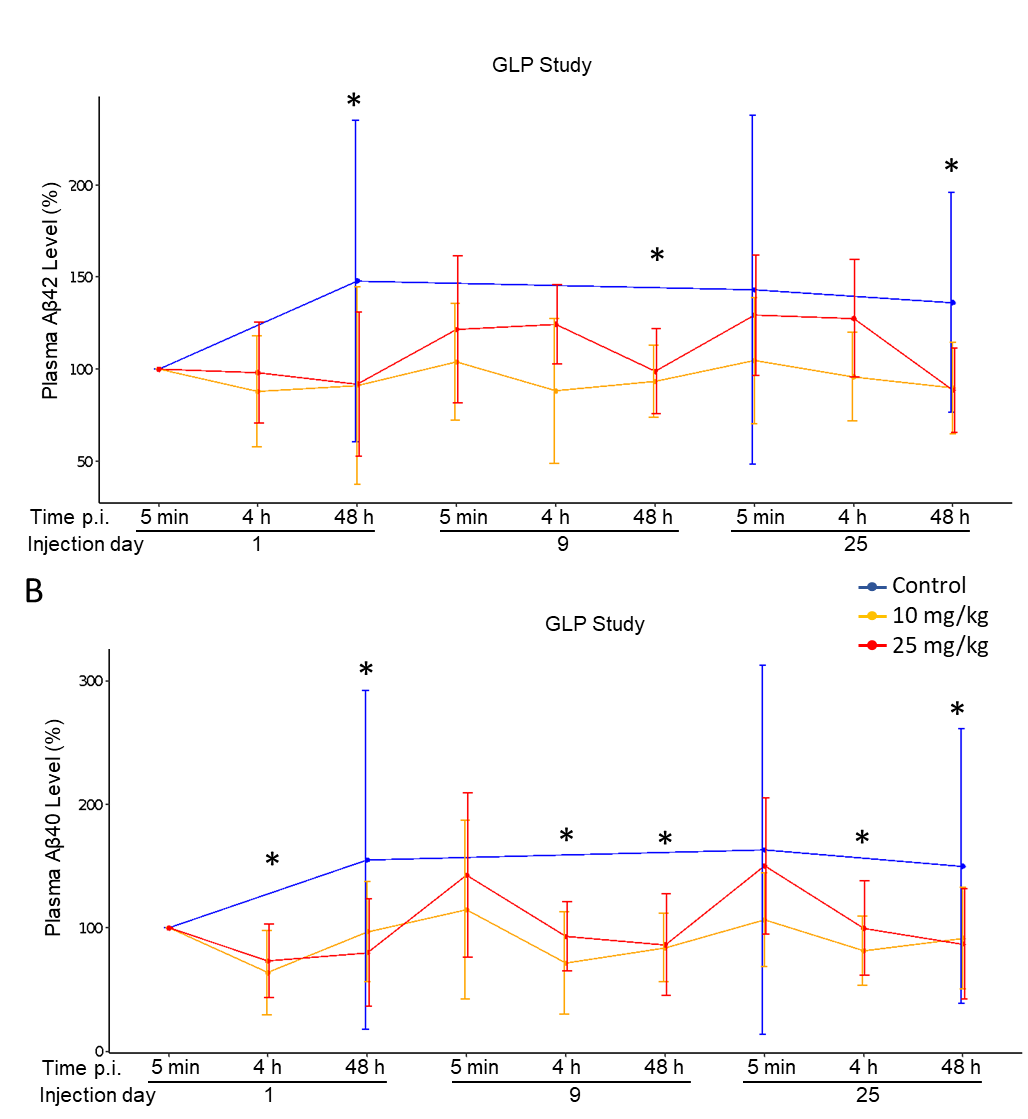


**Figure S1**: Plasma Aβ42 (A) and Aβ40 (B) levels following CS-6253 injection in the GLP study. A) The Aβ42 levels in plasma at 48 h was significantly lower compared to 5 m p.i. on the day of injection (p = 0.001) B) The effect on Aβ40 levels in plasma at 4 h and 48 h were significantly lower compared to 5 m p.i. on the day of injection (p < .001 for both). The values were shown as percent change from the first measurement point at 5 min p.i. on day 1. The measurements in the placebo arm were only done at 5 min and 48 hours on days 1 and 25. The mixed effects models for this measurement used only actively treated animals; fixed effects included indicator variables for treatment dose, injection number, and time of assessment (4 hours and 48 hours, each compared to the 5-minute baseline timepoint).


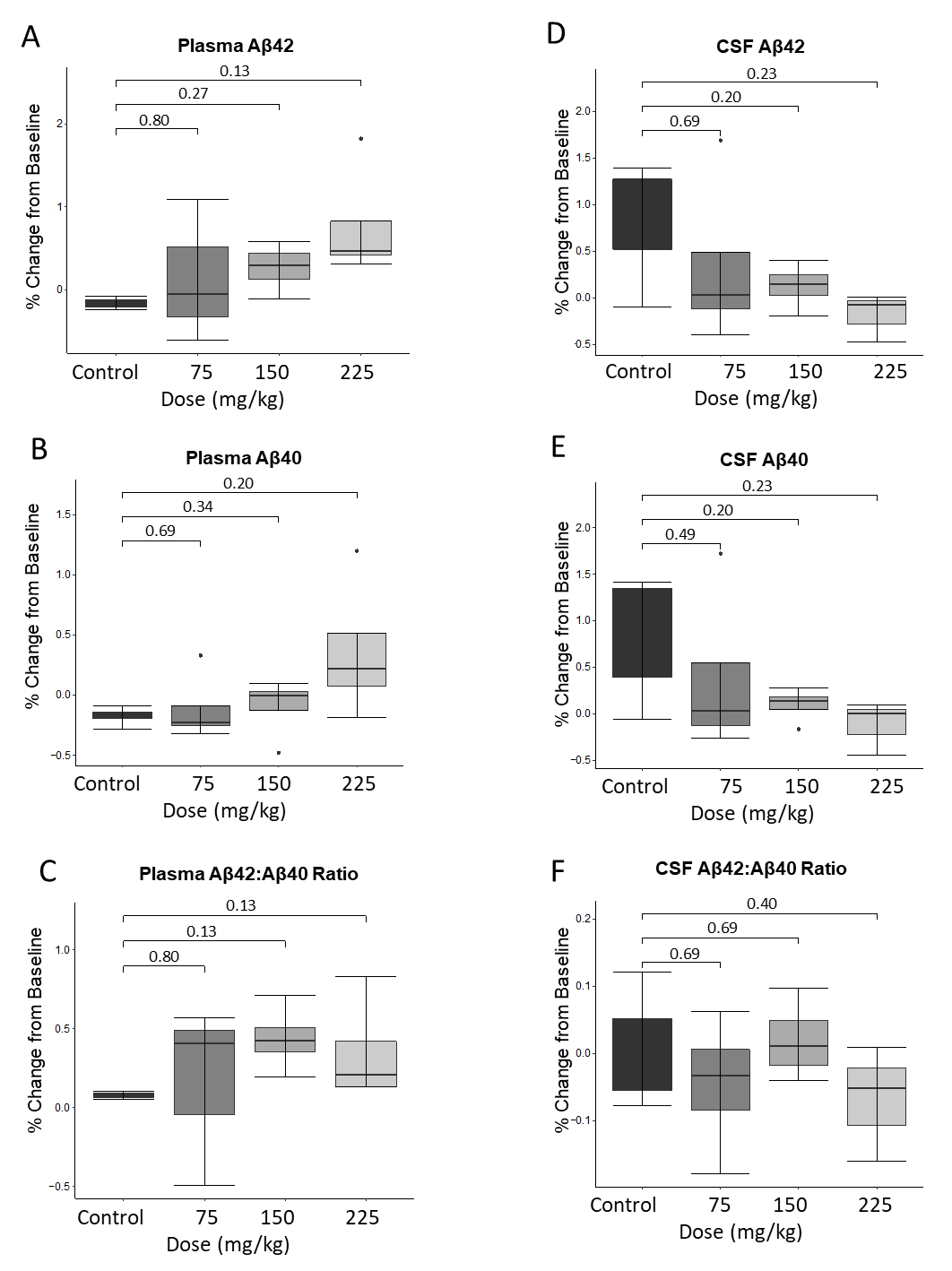


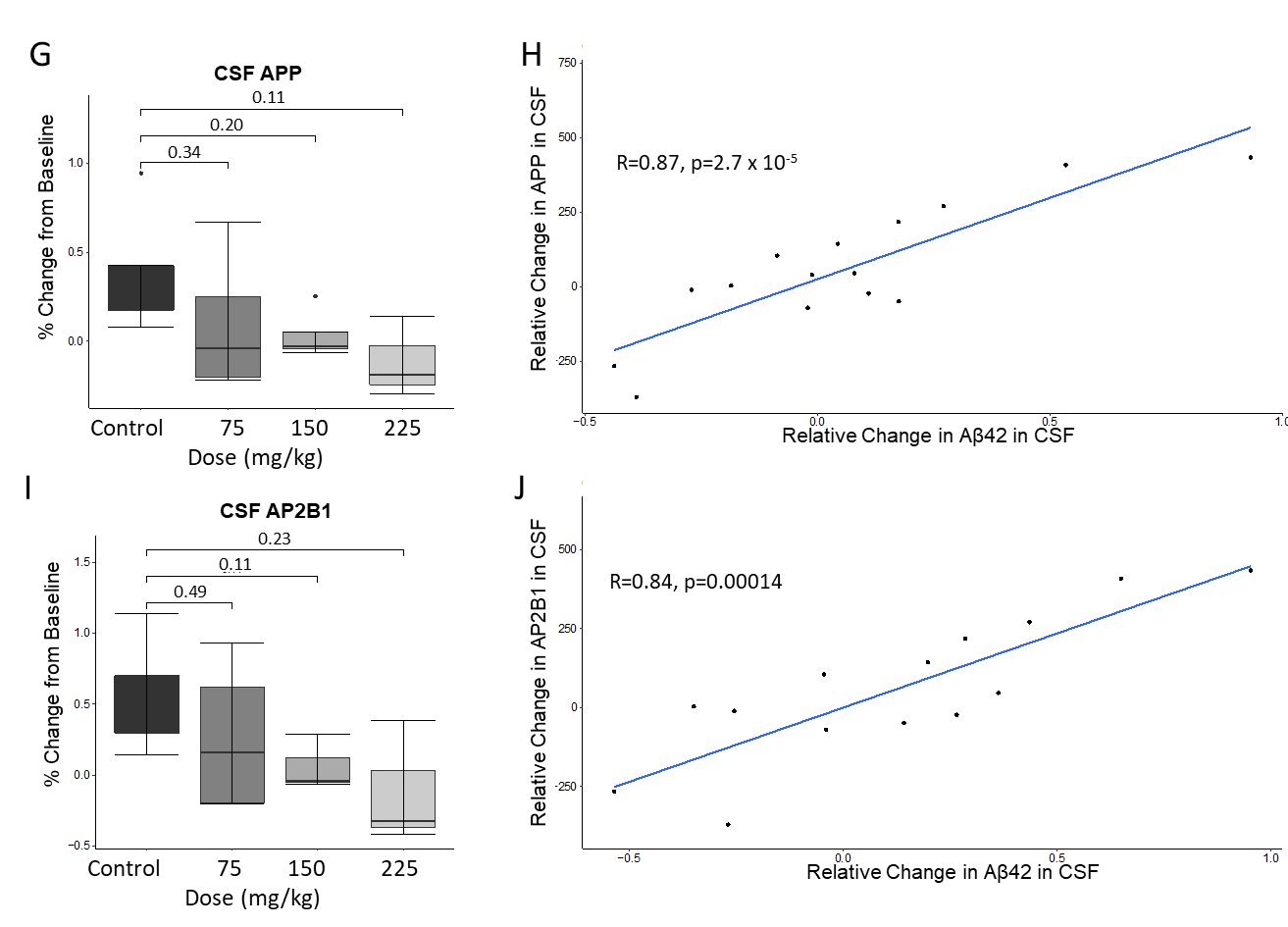


**Figure S2**: DRF study plasma and CSF Aβ42, Aβ40, and Aβ42/40 and CSF APP and AP2B1 levels. A and B) Both Aβ42 and Aβ40 levels were non-significantly increased in the plasma of DRF animals. C) The increase in plasma Aβ42/40 ratio was also not significant. D and E) Aβ42 and Aβ40 levels in the CSF as well as F) the plasma Aβ42/40 ratio were not significantly different. G) Although not significant CS-6253, decreased CSF APP levels. I) Similarly, a trend for decreased AP2B1 levels were observed but the changes were not significant. H and J) Changes in CSF APP levels (H) and AP2B1 (J) correlated with the change in Aβ42 level (R=0.87, p < 0.001 and R=0.84, p < 0.001 respectively) The values were shown as percent change from the baseline measurement. The group comparisons were obtained using wilcoxon signed-rank test, and the correlation plots using Pearson correlations.


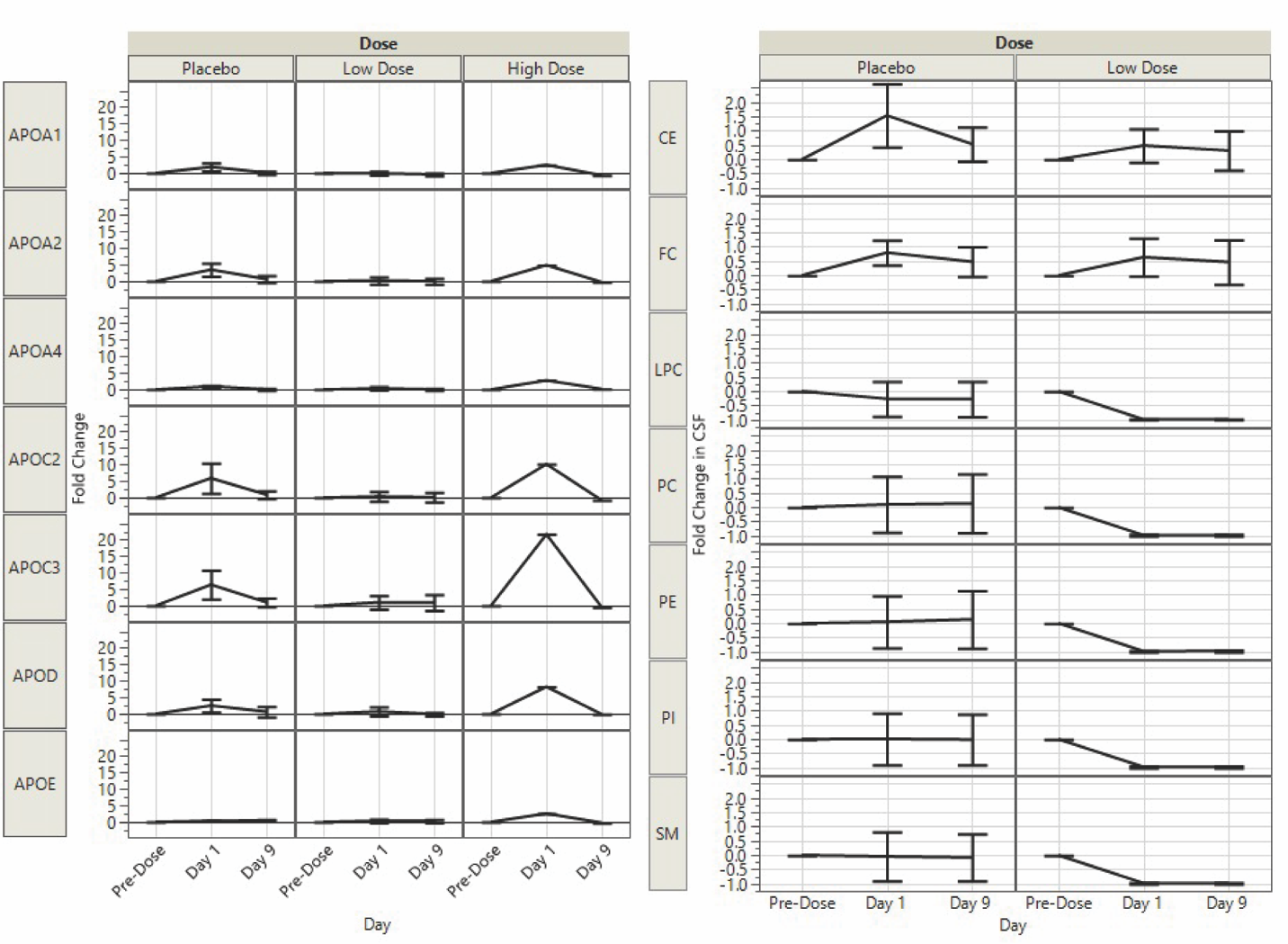


**Figure S3:** Profiles of certain types of CSF apolipoprotein and lipid levels after CS6253 injections at 75 mg/kg (low dose) and 150 mg/kg (high dose) in the DRF study. Fold change for treatment groups were plotted against various injection days. Fold change as a ratio was calculated by first taking the difference between the single measurement of an analyte of a subject at a specific time-point from its mean value in all subjects of the placebo group across all time points, then divided by that mean value. Error bars represent standard deviation. CE: cholesteryl esters, FC: free cholesterol, LPC: Lysophosphatidylcholine, PC: Phosphatidylcholine, PE: Phosphatidylethanolamine, PI: phosphatidylinositol, and SM: sphingomyelin.


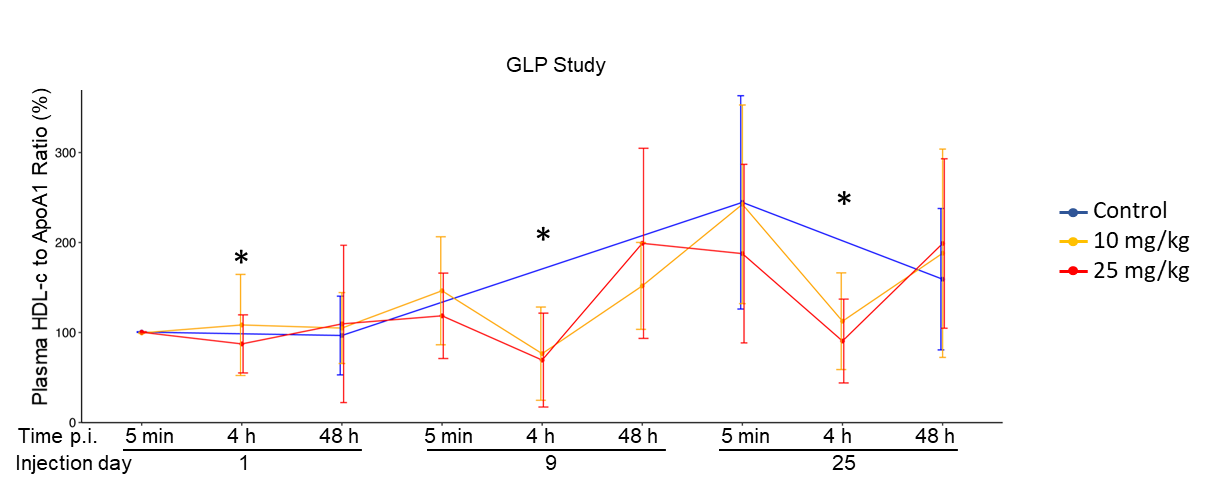


**Figure S4**: Plasma HDL-cholesterol to ApoA1 ratio in GLP study. The decrease was significant at 4 h p.i compared to 5 m p.i. on the day of injection (p < 0.001). The values were shown as percent change from the first measurement point at 5 min p.i. on day 1. The measurements in the placebo arm were only done at 5 min and 48 hours on days 1 and 25. The measurements in the placebo arm were only done at 5 min and 48 hours on days 1 and 25. The mixed effects models for this measurement used only actively treated animals; fixed effects included indicator variables for treatment dose, injection number, and time of assessment (4 hours and 48 hours, each compared to the 5-minute baseline timepoint).


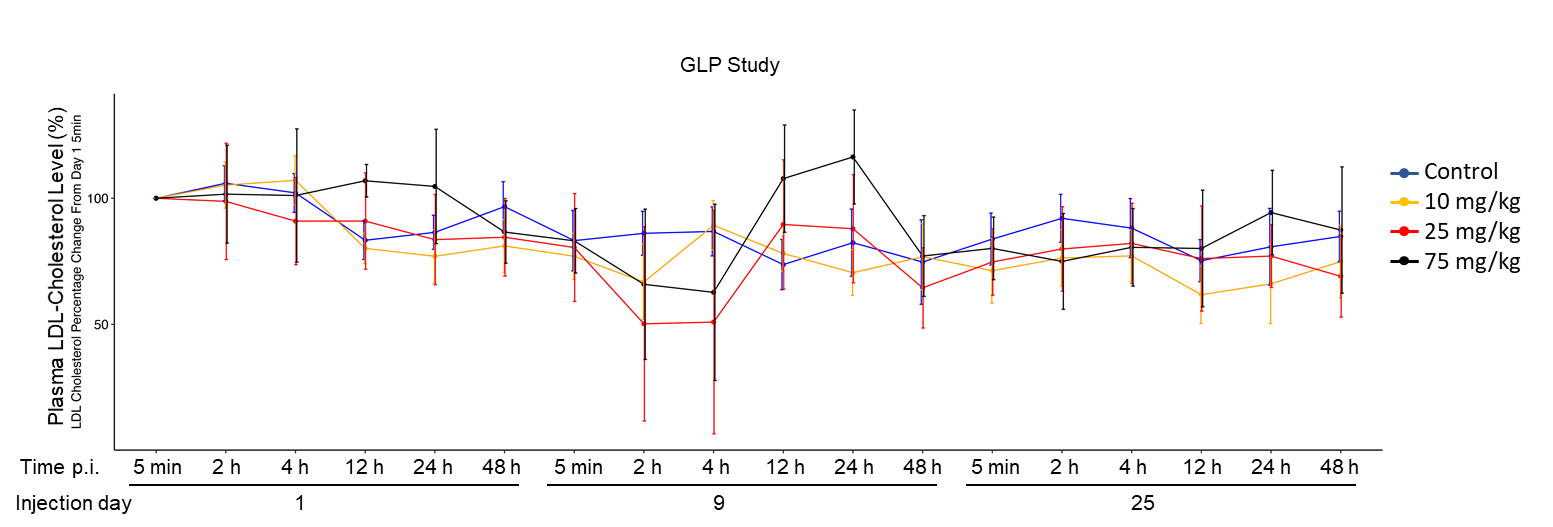


**Figure S5**: Plasma LDL-cholesterol levels did not change significantly in GLP study. The values were shown as percent change from the first measurement point at 5 min p.i. on day 1. The analysis was done using mixed effects model, with cholesterol levels modeled as a function of fixed effects including treatment (active compared to placebo), and indicator variables for hours since injection (i.e., time of injection) and injection number, and total time under study; a random intercept of subject was specified to model correlated outcomes arising from repeated measurements.


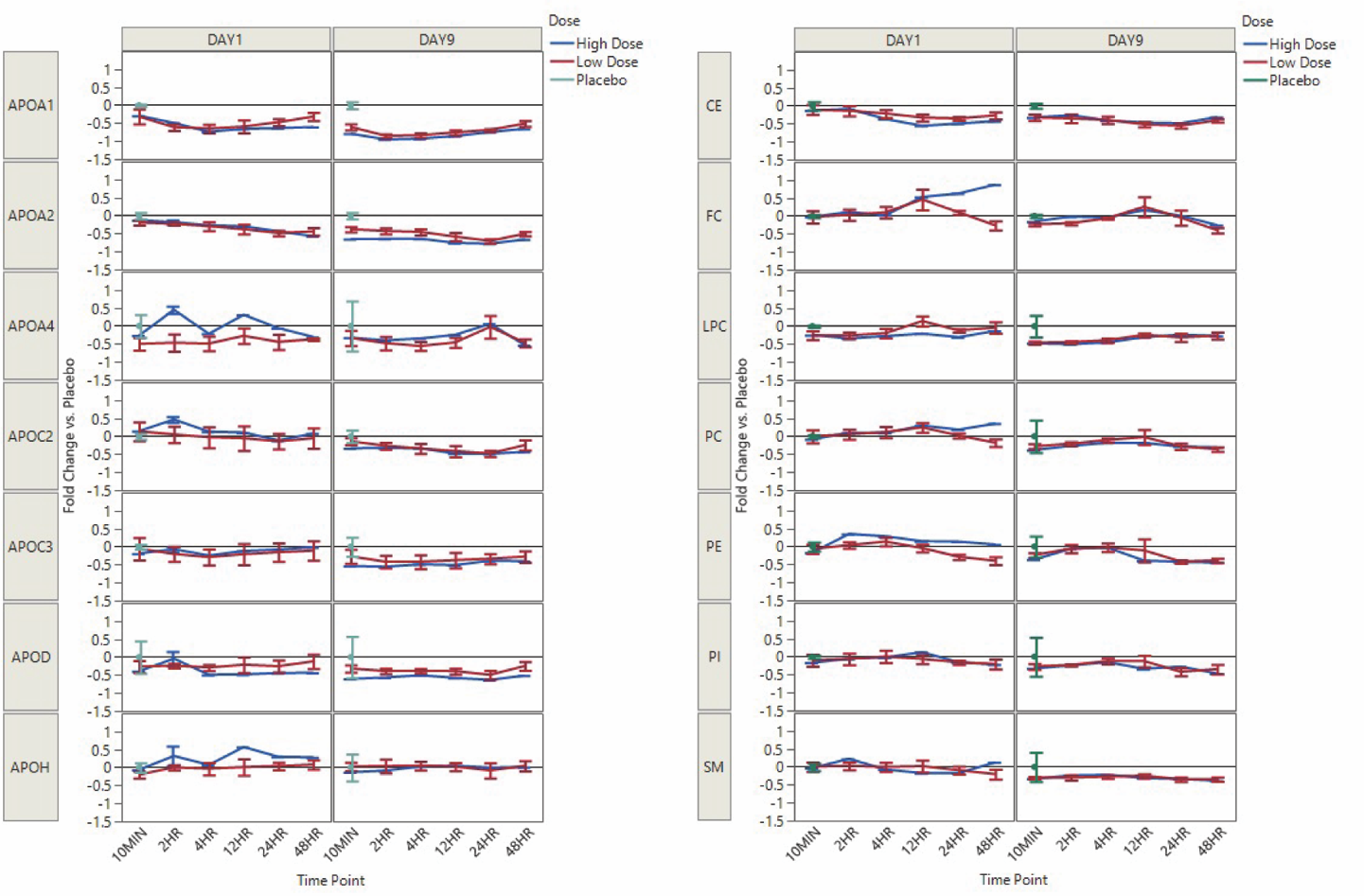


**Figure S6:** Profiles of certain types of plasma apolipoprotein and lipid levels after CS6253 injections at 75 mg/kg (low dose) and 150 mg/kg (high dose) in the DRF study. Levels of most of the measured apolipoprotein and lipids did not change significantly. Cholesteryl ester levels seem to decrease after the first two time points. Fold change for treatment groups were plotted against various injection time points. Fold change as a ratio was calculated by first taking the difference between the single measurement of an analyte of a subject at a specific timepoint from its mean value in all subjects of the placebo group across all time points, then divided by that mean value. Placebo measurements were only taken 10 minutes after injection on day 1 and day 9. Error bars represent standard deviation. CE: cholesteryl esters, FC: free cholesterol, LPC: Lysophosphatidylcholine, PC: Phosphatidylcholine, PE: Phosphatidylethanolamine, PI: phosphatidylinositol, and SM: sphingomyelin.


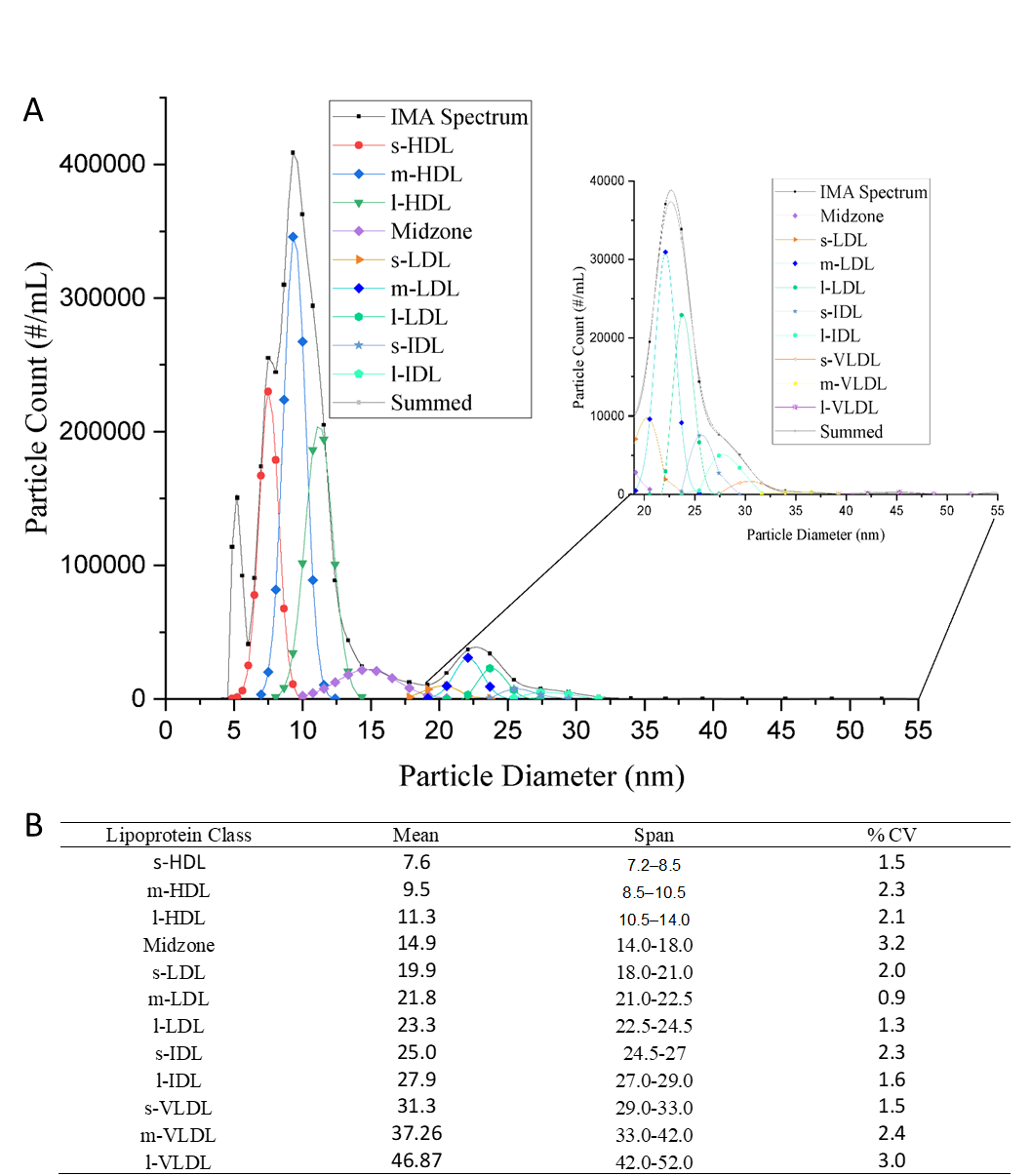


**Figure S7**: A) Ion-mobility spectrum of pre-injection monkey plasma lipoproteins’ profile distribution analyzed by Voigt probability distribution curves (R^2^ = 0.998). B) Summary of measured lipoprotein sizes measured by ion-mobility and their spans.


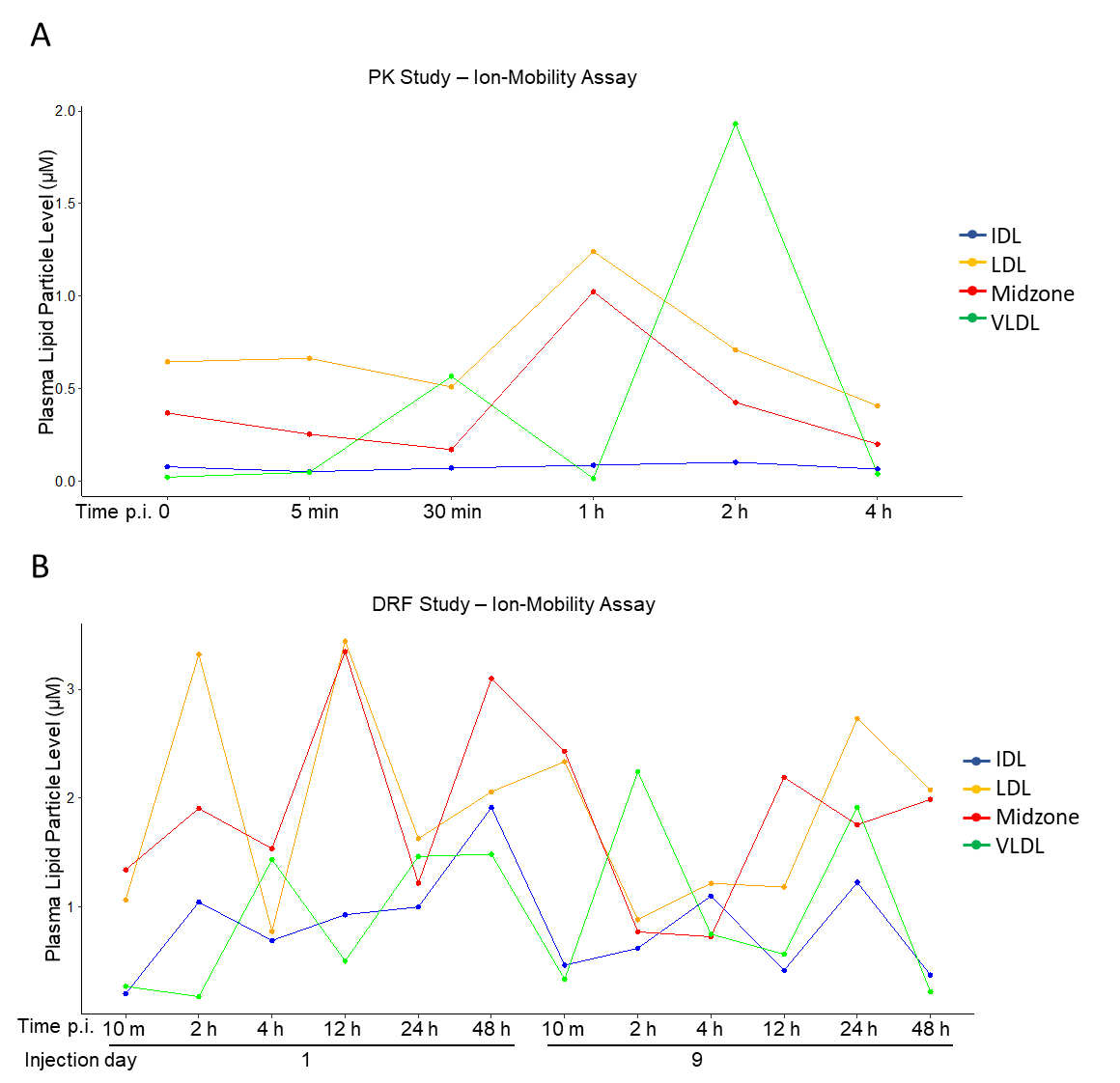


**Figure S8**: Analysis of IDL, LDL, Midzone, and VLDL particles A) for one monkey in the PK study and B) for one monkey in the DRF study. Particle concentrations in the plasma were calculated using ion-mobility analysis.


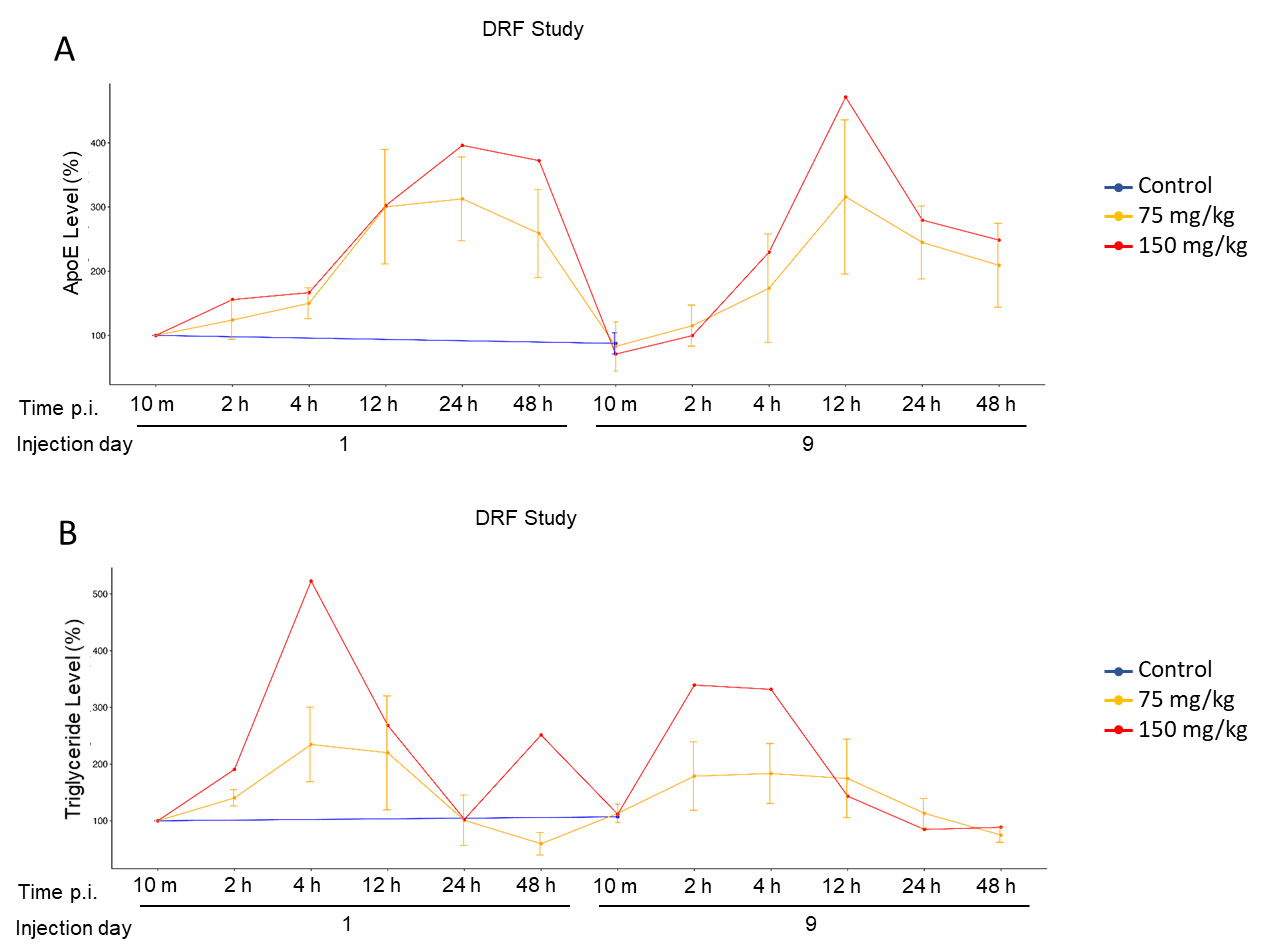


**Figure S9**: A) ApoE and B) total triglyceride levels analyzed by mass spectrometry for two monkeys in the DRF study follow a similar trend to our earlier analysis.


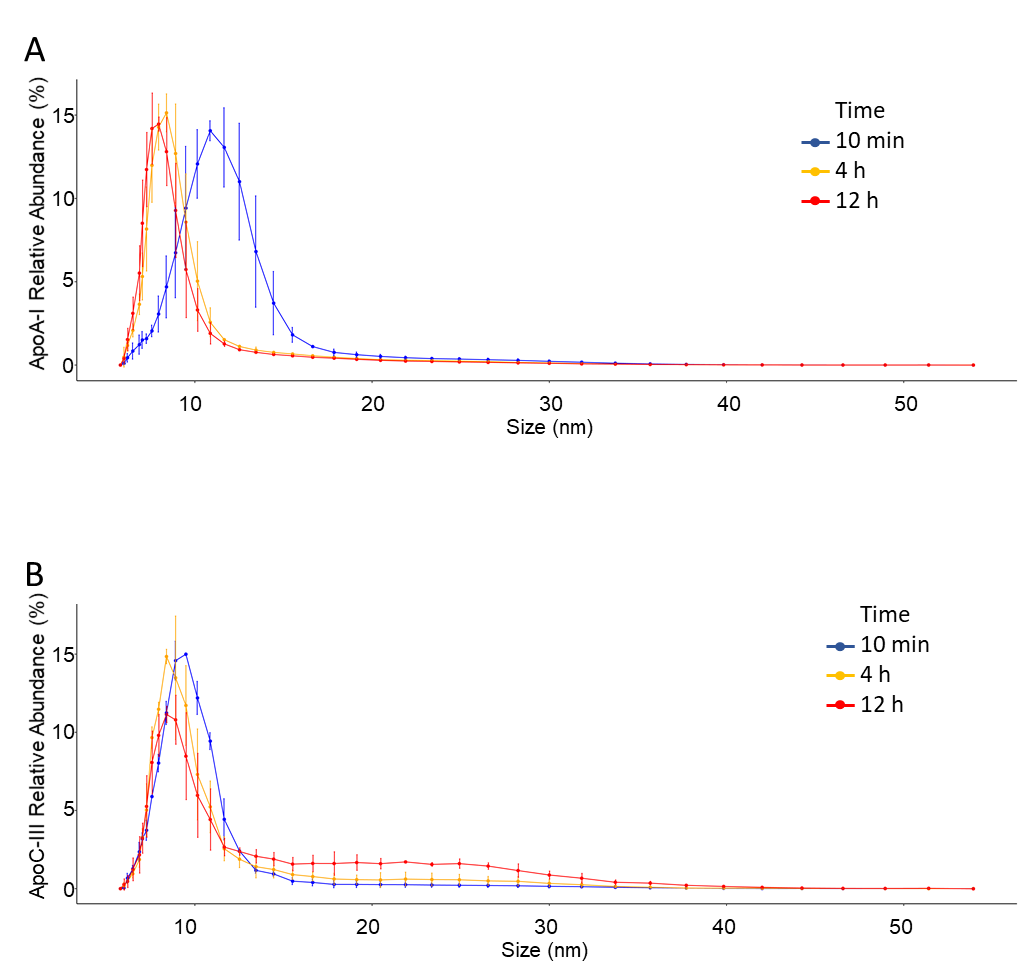


**Figure S10**: Analysis of abundance of A) apoA-I and B) apoC-III in HDL, IDL and LDL particles using AF4 for two monkeys in the DRF study.

| **Study Group** | **PK Study** | **Dose-range finding (DRF) Study** | **GLP Study (Study 1478.01)** |
| --- | --- | --- | --- |
| Cynomolgus monkeys: n= sex / age / weight | 2 male / 8 years old / 5 kg | 8 male & 8 females / 2 years, 3 months – 2 years, 10 months / 2.0 – 2.6 kg | 9-16 male & 9-16 female / 2-4 years old / males: 2.19 to 2.5 kg, females: 2.14 to 3.04 kg |
| Dosing (iv injection) regimen | single dose, 25 mg/kg | Plc, 75, 150, 225 mg/kg, q.a.d. for 11 days (5 injections) | Plc, 10, 25, 75 mg/kg q.a.d. for 30 days (15 injections) |
| PK (PD) curve start | Day 1 | Days 1 and 9 (5^th^ injection) | Days 1, 9, 25 |
| Tissue collection | Plasma and CSF  Before & after injection | Plasma and CSF  Before & after 1^st^ and 5^th^ injection | Plasma  After 1^st^, 5^th^ & 13^th^ injection |
| PK: CS-6253 half-life | 20h | 12-16 h | 11-19h |

**Table S1:** The details of the design of each study.

| Variable | Time | Groups | Effect Size* | N per Group |
| --- | --- | --- | --- | --- |
| Plasma Aβ42:40 | At 48 h p.i. (on any day) | 10mg/kg vs. placebo | 0.43 | 68 |
|  |  | 25mg/kg vs. placebo | 0.40 | 77 |
| CSF AP2B1 | Baseline to Day 9 (Percentage Change) | 75mg/kg vs. placebo | 0.57 | 40 |
|  |  | 150mg/kg vs. placebo | 1.45 | 7 |
|  |  | 225 mg/kg vs. placebo | 1.54 | 7 |
| CSF Aβ42 | Baseline to Day 9 (Percentage Change) | 75mg/kg vs. placebo | 0.58 | 37 |
|  |  | 150mg/kg vs. placebo | 1.30 | 9 |
|  |  | 225 mg/kg vs. placebo | 1.95 | 5 |

**Table S2:** The power calculations for selected variables that showed a trend change without statistical significance. * Cohen’s effect size.

**Table S3**: Concentrations and percent distributions of s-, m-, and l-HDL particles in one monkey’s plasma in the PK study.
